# Bias in Medical AI in Relation to Patient Empowerment and Digital Equity: Scoping Review

**DOI:** 10.2196/94439

**Published:** 2026-11-09

**Authors:** Lisa-Maria Norz, Elizaveta Beeck, Elske Ammenwerth

**Affiliations:** 1 Department for Biomedical Computer Science and Mechatronics, Institute of Medical Informatics, UMIT TIROL - Private University for Health Sciences and Health Technology, Eduard-Wallnöfer-Zentrum 1, Hall i. Tirol, 6060, Austria, 43 6642095599

**Keywords:** AI, medical AI, patient empowerment, digital divide, digital equity, algorithmic bias, social bias, design bias, structural bias, health care AI, intersectionality

## Abstract

**Background:**

AI is increasingly embedded in health care and digital health applications, shaping clinical processes as well as how patients access information and participate in care. Research on bias in medical AI has largely focused on technical and performance-related disparities. Less attention has been paid to how such biases may affect patients’ access, understanding, trust, and opportunities for meaningful involvement in care.

**Objective:**

This scoping review aimed to map how studies of bias in medical AI describe patient-related consequences relevant to patient empowerment and the digital divide and to identify gaps across technical, social, structural, and patient-centered perspectives.

**Methods:**

This scoping review followed JBI (Joanna Briggs Institute) methodology and PRISMA-ScR (Preferred Reporting Items for Systematic Reviews and Meta-Analyses extension for Scoping Reviews) reporting guidance. PubMed/MEDLINE and EBSCOHost databases were searched in August 2025 and supplemented by Google Scholar searches. Peer-reviewed studies published from 2015 onward were included if they addressed bias in medical AI and described patient-related consequences relevant to patient empowerment or digital inequality. Data charting focused on AI application types, bias dimensions, patient empowerment domains, digital divide–related factors, intersectionality, and debiasing strategies. Findings were synthesized descriptively and thematically.

**Results:**

The search identified 497 records, of which 22 studies met the inclusion criteria. Bias was represented as a multidimensional sociotechnical phenomenon, with social bias identified most frequently (21/22 studies), followed by algorithmic bias (16/22 studies), structural bias (14/22 studies), and design-related bias (12/22 studies). Patient empowerment was most frequently addressed through involvement in care (n=13), followed by health literacy (n=9) and trust and assurance (n=7), whereas broader psychosocial and everyday-life domains were addressed less frequently. Digital divide–related factors were identified in 19 of the 22 studies, with digital literacy and usability barriers reported most frequently. Seven studies considered 2 or more social or structural dimensions of disadvantage. Although many studies discussed approaches that could mitigate bias-related barriers, explicit empirical evaluation of debiasing strategies in relation to patient empowerment or digital equity was largely absent.

**Conclusions:**

Patient-related consequences of bias in medical AI extend beyond algorithmic performance and are reflected in access, understanding, trust, and involvement in care. Broader empowerment dimensions, multidimensional disadvantage, and empirically evaluated mitigation strategies remain comparatively underexplored.

## Introduction

### Medical AI and Bias in Health Care

AI is rapidly transforming health care by reshaping clinical workflows, diagnostics, patient-provider communication, and health-related decision-making [1-3]. AI-based systems are now embedded in a wide range of digital health applications, including medical imaging, clinical decision support, triage systems, symptom checkers, conversational agents, and patient education tools [4,5], thereby directly influencing how patients access information, experience care, and participate in health decisions [6,7].

While definitions of AI vary across disciplines, we adopt the widely used definition of AI as computer systems capable of performing tasks that typically require human intelligence, such as reasoning, learning, perception, and language understanding [8]. In this scoping review, we focus specifically on medical AI, understood as the application of AI-based systems within health care settings to support clinical processes, patient care, and health-related decision-making, spanning both clinician-facing and patient-facing tools [9]. This definition encompasses machine learning (ML) models, large language models (LLMs), and rule-based systems that process health data, generate recommendations, or interact with patients through digital interfaces.

In this scoping review, the term “medical AI” is used as the primary umbrella term to describe AI-based systems applied within health care contexts, including patient-facing and clinician-facing applications.

A growing body of evidence highlights the potential benefits of medical AI for health care quality and accessibility. Empirical studies suggest that AI-based tools can improve diagnostic accuracy [10,11], reduce clinical workload [4], enhance patient education and comprehension [12], and provide more timely, personalized, and scalable information [12,13]. Taken together, these developments suggest that medical AI holds considerable promise for improving efficiency, quality of care, and access to health services.

At the same time, there is increasing concern that AI systems in health care may introduce, perpetuate, or amplify risks related to fairness, transparency, accountability, and inclusiveness. Although medical AI is often framed as a novel technological development, many of the challenges associated with its implementation are not new. Rather, they are deeply rooted in longstanding structural inequalities, social determinants of health, and historical patterns of exclusion within health care systems [3,14]. Empirical studies have shown that algorithmic bias can lead to systematically different outcomes across population groups, particularly along dimensions such as race, gender, age, socioeconomic status, and language proficiency [14,15]. Recent work further indicates that biases in LLMs (including attribution and representational bias) are not merely theoretical concerns but may directly affect the quality, framing, and accessibility of patient information and clinical recommendations, with the potential to reinforce or widen existing health inequalities [3,16].

However, differential outcomes across population groups do not necessarily indicate differences in patient empowerment. In many studies, the effects of bias on patient empowerment are not examined directly but are instead inferred from proxy indicators such as access, system performance, or the quality of information provided [17].

### Theoretical Background

Much of the existing literature on medical AI has focused on clinician-centered outcomes, such as diagnostic performance, workflow optimization, or decision support for health care professionals [10,18]. In contrast, comparatively less attention has been paid to how medical AI systems shape patient roles, experiences, and agency in health care. This gap is increasingly problematic, as medical AI is not used only in clinical or professional contexts. Medical AI tools are now increasingly used in patient-facing contexts, such as chatbots, automated medical history-taking systems, digital symptom checkers, or AI-generated patient education materials [19-21]. These tools can directly influence how patients interpret symptoms, what options they perceive as viable, when they seek professional help, how they communicate with providers, and how they navigate complex care pathways.

As a result, medical AI has become deeply intertwined with the concept of patient empowerment in digital health. Patient empowerment encompasses dimensions such as shared decision-making, health literacy, disease management, autonomy, and digital participation [22,23]. In theory, medical AI has the potential to support empowered patients by improving access to information, facilitating self-management of chronic conditions, and enabling more informed and participatory care [24]. However, empowerment is neither an automatic nor a guaranteed outcome of AI adoption. Instead, whether AI supports or undermines patient empowerment depends on how these systems are designed, implemented, and embedded within existing social and institutional contexts [25].

A central challenge in studying patient empowerment is the absence of a universally accepted definition or measurement framework. Reviews have documented substantial conceptual and methodological heterogeneity, identifying dozens of definitions and instruments without reaching consensus [22,23]. Despite this diversity, the definition of Funnell et al [26] remains one of the most frequently cited and foundational conceptions. According to Funnell et al [26], empowerment refers to: “[…] the discovery and development of one’s inherent capacity to be responsible for one’s own life. People are empowered when they have sufficient knowledge to make rational decisions, sufficient control and resources to implement their decisions, and sufficient experience to evaluate the effectiveness of their decisions” (p. 38).

Building on this theoretical foundation, this scoping review operationalizes patient empowerment using the thematic categorization proposed by Mora et al [23]. Empowerment-related findings are classified into patient-reported experiences (eg, trust and involvement in care) and patient-reported outcomes (eg, health literacy, health behaviors, perceived health, illness experiences, managing daily life, and social support). Rather than treating empowerment as a latent variable to be quantified, we conceptualize it as a set of necessary conditions that may be supported or constrained by medical AI systems.

Bringing these bodies of literature together, bias in AI affects not only diagnostic accuracy but also access, autonomy, and trust, which are core components of patient empowerment. Importantly, various forms of bias in medical AI may directly undermine patient empowerment by limiting access, distorting information, or excluding specific groups from meaningful participation [27,28]. These bias categories align with widely cited conceptual frameworks that distinguish between technical, social, and structural sources of bias in AI systems [27,28]. Technical biases, such as data imbalance or algorithmic optimization errors, can result in lower performance for underrepresented populations [14,29]. Social biases may reflect existing stereotypes or assumptions embedded in training data or design choices [14,30]. Structural biases arise when AI systems are deployed within health care infrastructures that already disadvantage certain groups, thereby risking the reinforcement of existing inequities [3,14].

These dynamics intersect closely with the digital divide, understood as disparities in access to digital technologies, connectivity, digital literacy, language proficiency, and the ability to meaningfully engage with digital health tools [23,31]. The digital divide is a multilayered concept: beyond mere access to devices or connectivity, it also encompasses differences in digital literacy, usage patterns, and the degree to which individuals can derive benefits from digital health services [32,33]. To link patient empowerment conceptually with digital divide and inclusion research, we draw on the foundational definition by Funnell et al [26] of empowerment as a process contingent on knowledge, control, and evaluative experience. From this perspective, digital inclusion is a prerequisite for empowerment, not a peripheral implementation issue. Digital divide dynamics can systematically undermine each of the core empowerment components by Funnell et al [26]: limited digital literacy, language discordance, or poor interface design constrains patients’ ability to acquire and comprehend knowledge; restricted access or usability barriers reduce patients’ control over decisions and resources; and unequal opportunities to interact with, question, or reflect on AI-supported recommendations limit patients’ capacity to evaluate the consequences of their decisions. Consequently, even when medical AI tools aim to empower patients, digital inequalities can limit this empowerment to a superficial form of participation.

Research on digital health interventions suggests that less privileged populations often initiate and continue the use of digital health tools at lower rates than more advantaged groups, leading to unequal opportunities for meaningful participation and agency [33]. Furthermore, an intersectional perspective is crucial, as patients often experience overlapping forms of disadvantage based on age, gender, ethnicity, socioeconomic status, disability, or migration background: factors that collectively shape how AI systems are experienced and whether empowerment is facilitated or hindered.

Beyond differences in access, research on digital inequality shows that benefits from digital health technologies depend on users’ skills, patterns of use, and resulting outcomes [33]. Following van Dijk [31], the digital divide can therefore be understood as a multilevel process involving unequal access, unequal capabilities, and unequal returns (rather than a single “divide”). In parallel, the concept by Heeks [32] of “adverse digital incorporation” highlights that inclusion into digital systems can be harmful when it occurs under unequal conditions, leading to new forms of dependency, surveillance, or reduced agency. For medical AI, this implies that patient-facing tools may not only exclude certain groups but may also involve patients in ways that undermine their autonomy, trust, and sense of control, which are core dimensions of patient empowerment. Importantly, these inequalities are often not external to AI systems but are shaped by design choices and deployment contexts, linking the digital divide closely to issues of design and structural bias.

Recent literature reviews have increasingly addressed fairness, bias, and inclusion in medical AI from different disciplinary perspectives. Existing reviews have examined demographic disparities and bias in medical LLMs, highlighting unequal performance and representation across patient groups and emphasizing the need for fairness-oriented mitigation strategies [34]. Other reviews have focused more broadly on bias recognition and mitigation approaches in health care AI, including algorithmic fairness, data imbalance, and governance-related challenges [35]. In parallel, reviews on digital health inequalities have emphasized that intersectional dimensions of disadvantage remain insufficiently addressed in digital health research, despite their importance for understanding unequal access, participation, and outcomes [36]. Recent work has further highlighted inclusivity challenges in AI-supported health care and social care systems, particularly regarding the underrepresentation and differential treatment of marginalized populations [37]. However, despite this rapidly growing body of literature, existing reviews remain fragmented across technical fairness research, digital health inequality research, and patient-centered health care perspectives. Most reviews focus either on technical aspects of algorithmic bias, on governance and mitigation strategies, or on broad ethical implications of AI in health care. Comparatively little integrative work has systematically examined how different forms of bias in medical AI relate specifically to patient empowerment and the digital divide, particularly in patient-facing applications and among intersectionally disadvantaged populations. By synthesizing technical, social, structural, and empowerment-related dimensions of bias within a single conceptual framework, this scoping review addresses an important gap at the intersection of medical AI, digital inclusion, and patient-centered care. In doing so, the review extends existing fairness-oriented medical AI literature beyond technical performance considerations alone by explicitly examining how bias relates to patient empowerment and digital inequality in patient-facing health care contexts.

### Research Problem

Despite rapidly growing interest in fairness, ethics, and equity in medical AI, existing research remains fragmented across disciplines, application areas, and outcome measures. To date, little integrative work has been carried out to systematically map types of bias in medical AI and examine how these biases relate to patient empowerment and the digital divide in health care, particularly in patient-facing applications and digitally mediated care. To address this gap, the present scoping review aims to systematically identify, analyze, and map the scientific literature on bias in medical AI, with a specific focus on its implications for patient empowerment and digital inclusion. By synthesizing evidence across technical, social, and structural dimensions of bias and situating these findings within an empowerment-oriented framework, this review seeks to provide a conceptual foundation for future research, responsible AI development, and equitable implementation of AI in health care. Consistent with the aims of a scoping review, this study prioritizes conceptual mapping and synthesis over causal inference or quantitative effect estimation.

### Research Question

This scoping review aims to answer the following question:

Which types of bias are described in scientific literature in relation to the use of medical AI in health care, and how are these biases described in relation to patient empowerment and the digital divide, particularly among vulnerable or intersectionally disadvantaged patient groups?

To further structure the analysis, the following subquestions were considered: (1) Which technical, social, or structural forms of bias are reported across different applications in medical AI in health care? (2) How is patient empowerment conceptualized and addressed in studies examining bias in medical AI?

## Methods

### Protocol and Registration

This scoping review was conducted to systematically identify, map, and synthesize the existing literature on bias in medical AI applications in health care, with a particular focus on patient empowerment and the digital divide. Given the exploratory nature of the research question and the conceptual heterogeneity of the field, a scoping review methodology was considered appropriate.

The review was conducted in accordance with the JBI (Joanna Briggs Institute) methodology for scoping reviews and is reported following the PRISMA-ScR (Preferred Reporting Items for Systematic Reviews and Meta-Analyses extension for Scoping Reviews). The completed PRISMA-ScR checklist is provided in Checklist 1.

As there is currently no dedicated registry for scoping reviews comparable to PROSPERO (International Prospective Register of Systematic Reviews) for systematic reviews, the review protocol was prospectively registered on the Open Science Framework (OSF) to ensure transparency and reproducibility and is available online [38]

### Eligibility Criteria

The research question and eligibility criteria were structured using the PCC (population-concept-context) framework, as recommended for scoping reviews by the JBI.

Regarding population (P), the population of interest includes individuals or population groups who may be affected by bias in the context of medical AI applications. Particular attention was given to marginalized or intersectionally disadvantaged groups, including but not limited to individuals differentiated by age, gender, ethnicity, socioeconomic status, disability, language proficiency, or migration background.

Regarding concept (C), the core concept of this review comprises different forms of bias associated with the use of medical AI, including technical, algorithmic, social, structural, and intersectional biases. In addition, the review examines how these bias types are described in relation to patient empowerment and the digital divide.

Regarding the context (C), the context included applications of medical AI across the patient journey, such as clinical decision support systems (CDSS), diagnostic tools, symptom checkers, conversational agents, LLMs, remote monitoring systems, and AI-based patient education tools. No restrictions were placed on geographic regions or health care systems.

Eligibility was determined to be aligned with the PCC framework. An overview of all inclusion and exclusion criteria is provided in Table 1.

Studies were considered eligible when they addressed patient-related implications of bias in medical AI either explicitly or implicitly. Explicit relevance included direct discussion of patient empowerment, participation, or digital inclusion, whereas implicit relevance referred to studies describing patient-facing consequences such as access barriers, usability challenges, differential comprehension, trust, or unequal benefit from AI-supported health care. Purely technical studies without discussion of patient-related, ethical, or social implications were excluded.

**Table 1.** Inclusion and exclusion criteria.

Criterion	Inclusion	Exclusion
Topics	Studies addressing bias, inequity, inequality, or discrimination in relation to medical AI in health care	Bias or inequality in health care without reference to AI
Relation to patient empowerment or digital divide	Explicit or implicit discussion of patient empowerment, participation, health literacy, digital inclusion, or digital divide	No relation to patient empowerment, participation, or digital access
Population	Focus on patients or patient groups, including vulnerable or marginalized populations	Exclusive focus on developers, providers, or organizations without patient perspective
Bias types	Technical, algorithmic, social, structural, or intersectional bias	Purely technical evaluations without social or ethical implications
Context or applications	Medical AI applications in health care (eg, diagnostics, CDSS^a^, monitoring, chatbots, LLMs^b^)	AI applications outside health care (eg, industry, education)
Publication type	Peer-reviewed journal papers (original studies, systematic, scoping, or narrative reviews)	Editorials, commentaries, opinion pieces without systematic methodology; gray literature
Language	English or German	Other languages
Publication period	Published from 2015 onward	Published before 2015

a CDSS: clinical decision support system.

b LLM: large language model.

### Information Sources

A structured literature search was conducted across the following databases in August 2025 to capture relevant medical, social science, and technical literature: PubMed/MEDLINE and EBSCOHost (including MEDLINE; APA PsychInfo; APA PsycArticles; ERIC; CINAHL Ultimate; Academic Search Elite; and additional social science and information science databases). Google Scholar was used as a supplementary source to identify additional relevant publications. The selection of databases was guided by the interdisciplinary scope of the review, aiming to capture biomedical, psychological, and social sciences perspectives on medical AI, bias, and patient-related outcomes. Together, PubMed/MEDLINE and EBSCOHost databases provide broad coverage of clinical, behavioral, and health services research relevant to the study objectives.

### Search

The search strategy was developed based on an initial exploratory search and iteratively refined. Search terms were adapted to the specific syntax of each database (Multimedia Appendix 1). The core search strategy combined terms related to medical AI, bias and equity, patient empowerment, and used Boolean operators:

(“artificial intelligence” OR “machine learning” OR “natural language processing” OR “deep learning” OR “clinical decision support” OR “healthcare AI” OR “large language model*” OR LLM* OR ehealth OR “e-health” OR telehealth OR “tele-health”)

AND

(bias* OR inequit* OR inequal* OR discriminat* OR fair* OR intersectional*)

AND

(“patient empowerment” OR “shared decision*” OR “patient participation” OR “patient inclusion” OR “patient engagement” OR “patient involvement”)

The search strategy was designed around the intersection of medical AI, bias and equity, and patient-related implications. Given the conceptual breadth and interdisciplinary nature of the topic, the strategy was iteratively refined during pilot searching to improve sensitivity to studies addressing patient-related consequences of bias. Limitations in the breadth of the search, including the databases covered and the potential omission of relevant studies using different terminology, are acknowledged in the Limitations section.

The initial search strategy included an explicit digital divide component. During pilot searching, this approach was found to be overly restrictive and risked excluding studies in which digital divide-related aspects were addressed implicitly rather than as a primary analytical category. The search strategy was therefore iteratively refined to better capture the contextual breadth of the field. All records identified in the initial search were retained for screening and synthesis. Further details regarding these adaptations are provided in the section “Deviations From the Registered Protocol.”

The reporting of the literature search was guided by the PRISMA-S (Preferred Reporting Items for Systematic Reviews and Meta-Analyses literature search extension) for search reporting. Full search strategies for all databases, including Boolean operators, database-specific syntax adaptations, and search limits, are provided in Multimedia Appendix 1 to enhance transparency and reproducibility.

### Selection of Sources of Evidence

All identified records were imported into Rayyan (Rayyan Systems, Inc) [39] for screening and management. Duplicate records were removed before screening.

Title and abstract screening were conducted independently by 2 reviewers using a blinded screening approach. Each reviewer assessed all records against the predefined inclusion and exclusion criteria. Discrepancies between reviewers were resolved through discussion and consensus. Full-text screening was subsequently performed for all records deemed potentially eligible based on title and abstract screening, following the same independent and consensus-based procedure.

### Data Charting Process

For all included studies (Multimedia Appendix 2), data were charted manually using a standardized data charting form developed iteratively by the research team (Multimedia Appendix 3). The data charting form was pilot-tested on a subset of included studies and refined iteratively to improve clarity and consistency across reviewers. The charting focused on bibliographic characteristics, study context, AI application characteristics, and content relevant to bias, patient empowerment, and digital equity.

Systematic reviews were included as independent sources of evidence to capture how bias, patient empowerment, and the digital divide are conceptualized at an aggregate level. The primary studies within these reviews were not re-charted to avoid duplication and ensure methodological consistency.

The following variables were charted from each publication: bibliographic information (authors, year of publication, title, and journal); study characteristics (country, study type, area or clinical context, and research question); AI characteristics (type of AI and description); population characteristics (age, sex, disease area, ethnicity, subgroups); bias-related aspects (reported bias types); patient empowerment (presence and qualitative description); digital divide (presence and description); intersectionality (consideration of two or more social or structural dimensions of disadvantage); and debiasing strategies (whether mitigation approaches were described or recommended and whether they were implemented and empirically evaluated).

The data charting process was performed independently by 2 reviewers. Discrepancies were discussed and resolved by consensus to ensure consistency and transparency.

To enable systematic synthesis, charted variables were coded using predefined categorical schemes. Coding was developed a priori based on the literature and refined during the pilot charting process.

### Data Items

#### Medical AI

Medical AI applications were categorized according to their primary application type and functional role, adopting a function-oriented approach informed by the World Health Organization (WHO) classification framework for digital health interventions [40]. As the WHO framework is not specific to AI technologies, the classification was adapted to the characteristics of the applications represented in the included studies. Applications were grouped into the following categories: chatbot or conversational agent, LLM-based applications, diagnostic AI, decision support AI, robotic systems, surveillance system, other ML- or natural language processing (NLP)–based application, and no specific AI tool. Each study was assigned to one primary category based on the predominant characteristics and function of the investigated AI application.

#### Bias

Bias types were coded using a multidimensional category system, grounded in literature on fairness, ethics, and bias in AI, particularly in health care contexts (Textbox 1). Prior research has emphasized that bias in AI systems is not a purely technical phenomenon but emerges across multiple layers of sociotechnical systems, including data, algorithms, design choices, institutional settings, and human interaction [27,28,41].

Multiple bias categories could be assigned to a single study, reflecting the sociotechnical nature of medical AI systems, where technical, social, and psychological dimensions of bias frequently co-occur or interact.

Consistent with the JBI guidance for scoping reviews, no formal methodological quality appraisal or risk-of-bias assessment was conducted, as the aim was to map conceptualizations and patterns rather than to assess intervention effectiveness.

Textbox 1. **Category system for bias types.**

- Algorithmic bias: systematic errors or unequal performance arising from data imbalance, model optimization choices, or learning processes that disadvantage specific population groups [14,28].
- Social bias: reproduction of societal stereotypes, norms, or historical inequities embedded in training data, labeling practices, or implicit assumptions about users and patients [41,42].
- Design bias: biases introduced through system design decisions, interface structures, usability assumptions, or default settings that privilege certain users while marginalizing others [27,41,43].
- Structural bias: biases that arise when AI systems are deployed within health care infrastructures characterized by existing power asymmetries, resource inequalities, and institutional constraints, thereby reinforcing rather than mitigating health disparities [3,14,27].
- Psychological bias: cognitive, affective, and behavioral biases that shape how patients and clinicians perceive, trust, interpret, or rely on AI systems [44].

#### Patient Empowerment

Given the absence of a single standardized, validated instrument for measuring patient empowerment in the literature [23], we did not operationalize patient empowerment as a quantitative score or outcome measure. Instead, we applied a theory-informed qualitative classification approach. All included studies met the review’s inclusion criteria by addressing bias in medical AI with relevance to patient experiences, participation, access, or agency, either explicitly or implicitly. Accordingly, the aim of the empowerment-related analysis was not to determine whether patient empowerment was present, but rather to characterize how patient empowerment was conceptualized and addressed across studies. Therefore, for each study, we first identified whether and how the AI application enabled patients to exercise control, participation, or understanding in line with the foundational definition of patient empowerment proposed by Funnell et al [26]. Based on this assessment, empowerment-related aspects were subsequently thematically classified using the domains described by Mora et al [23]. This allowed us to systematically map which dimensions of patient empowerment are emphasized, underrepresented, or indirectly affected in studies on medical AI.

The following empowerment domains were applied (multiple coding allowed) and are summarized in Table 2.

This approach allowed for a nuanced mapping of how patient empowerment is conceptualized and operationalized across heterogeneous AI applications in health care.

**Table 2.** Categories of patient empowerment. Based on the categories by Mora et al [23].

ID	Category	Description
TA	Trust and assurance	Confidence in care, trust in systems or providers
IC	Involvement in care	Including shared decision-making
HL	Health literacy	Knowledge, comprehension, information accessibility
HB	Health behaviors	Adherence, self-care, self-management
PH	Perceived health	Quality of life, well-being
IPE	Illness perceptions and experiences	Emotional responses, illness understanding
MDL	Managing daily life	Autonomy, coping, problem-solving, functional ability
SS	Social support	Peer, family, community support

#### Digital Divide and Intersectionality

The digital divide aspects were coded using a theory-informed scheme, aligned with multilevel digital inequality models (eg, differential access, capabilities or digital literacy, and usage conditions), and were coded dichotomously (yes/no) and recorded when studies explicitly or implicitly described disparities related to access, digital literacy, language, infrastructure, or differential benefit from AI-based tools.

Intersectionality was coded as present when studies considered two or more social or structural dimensions of disadvantage (eg, age × gender, ethnicity × socioeconomic status) in relation to bias, patient empowerment, or digital access.

#### Debiasing Strategies

Debiasing strategies were documented qualitatively and coded according to whether mitigation approaches were described or recommended and whether they were implemented and empirically evaluated within the study.

### Synthesis of Results

Charted data were synthesized descriptively and thematically in accordance with scoping review methodology. Quantitative study characteristics, including study design, AI application type, clinical context, bias categories, empowerment domains, and digital divide dimensions, were summarized using frequencies and distributions.

Qualitative findings were analyzed using a theory-informed categorical approach based on the predefined coding framework for bias, patient empowerment, digital divide dimensions, and intersectionality. Particular attention was given to how studies conceptualized the relationships between AI-related bias, patient empowerment, and digital inclusion, including whether these relationships were addressed explicitly or implicitly.

To further examine patterns across the evidence base, bias categories, patient empowerment domains, and digital divide dimensions were mapped against medical AI application types. These descriptive cross-mappings were used to identify combinations that were comparatively frequent, sparsely represented, or absent within the included evidence base. The resulting patterns were visualized using heatmaps, with cell values representing the number of studies addressing each combination. Because multiple categories could be assigned to individual studies, counts were nonmutually exclusive. The mappings describe patterns of co-occurrence within the evidence base and were not interpreted as evidence of statistically tested associations. No inferential comparisons or assessments of evidence certainty were performed.

Because bias, empowerment, and digital divide dimensions frequently co-occurred within individual studies, multiple coding was permitted across all analytical categories. Accordingly, a single study could be assigned to more than one bias category, empowerment domain, or digital divide dimension where conceptually relevant. This approach reflects the multidimensional and sociotechnical nature of medical AI systems and enables the identification of overlapping patterns and thematic interconnections across the literature.

Findings were synthesized narratively and visually through thematic mapping, tables, and descriptive visualizations to identify recurring patterns, conceptual overlaps, and research gaps across the included studies. Consistent with the aims of a scoping review, the synthesis focused on mapping conceptualizations, patterns, and thematic relationships rather than evaluating intervention effectiveness or estimating pooled effects.

### Deviations From the Registered Protocol

This scoping review was conducted based on a protocol prospectively registered on the OSF [38]. During the review process, one adaptation had to be made to ensure the feasibility and conceptual relevance of the study, which is transparently reported here in line with best practices for scoping reviews.

The original search strategy included an explicit search block related to the digital divide. However, pilot searches revealed that this approach substantially limited the number of retrieved records and excluded studies in which digital divide-related aspects were addressed implicitly (eg, through bias, access, or usability considerations) rather than as a primary analytical focus. To avoid an overly restrictive search and to better capture the conceptual breadth of the field, the digital divide search block was removed. Importantly, all records identified in the initial search were retained and included in the screening process to ensure that explicitly relevant studies were not lost.

No other substantial deviations from the registered protocol were made. All other methodological procedures, including study selection, charting process, and synthesis, were conducted as specified in the protocol.

## Results

### Selection of Records of Evidence

Our literature search identified a total of 497 records, including 243 records from PubMed/MEDLINE and 254 records from EBSCOHost (see the PRISMA [Preferred Reporting Items for Systematic Reviews and Meta-Analyses] flow diagram, Figure 1). After removal of 139 duplicate records, 358 unique records remained. Google Scholar was used for supplementary searching, but no additional eligible records were identified.

In total, 358 records were then screened at the title and abstract level, where 250 records were excluded. A total of 17 records were excluded because they represented protocols or were otherwise not eligible for this scoping review (eg, not peer-reviewed). Further, 233 records were excluded because they did not focus on patients (n=137) or did not involve the use of AI-based tools in health care contexts (n=96).

A total of 107 records were sought for full-text retrieval, of which 4 could not be retrieved. Consequently, 103 full-text papers were assessed for eligibility. During full-text screening, 82 papers were excluded, primarily because the lack of patient focus became evident during full-text review (n=52), or the missing involvement of medical AI applications (n=29) became evident.

Overall, 22 studies met the inclusion criteria and were included in the final synthesis [7,12,19-21,45-61]. The study selection process is illustrated in the PRISMA flow chart (Figure 1).

**Figure 1.**
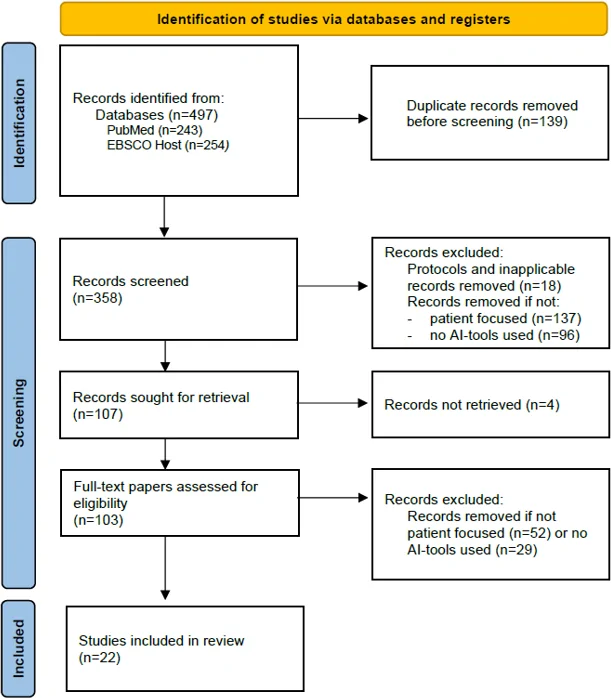
PRISMA (Preferred Reporting Items for Systematic Reviews and Meta-Analyses) flowchart of the literature search and study selection process.

### Characteristics of Included Studies

#### Overview

A total of 22 studies were included in the final synthesis. Table 3 shows an overview of the included studies’ characteristics. The included publications were predominantly published between 2016 and 2025, reflecting the recent and rapidly evolving nature of research on medical AI, bias, and patient-related outcomes. Studies originated mainly from North America, Europe, and Asia, with the United States (8/22, 36.4%) and European countries (8/22, 36.4%) most frequently represented, followed by Asian countries (5/22, 22.7%) and Canada (1/22, 4.5%).

Regarding study design, the literature comprised a heterogeneous mix of quantitative (n=12), qualitative (n=5), and mixed methods studies (n=4), alongside one systematic review. Quantitative designs most commonly included observational cohort studies, cross-sectional surveys, randomized controlled trials, and feasibility or usability evaluations, while qualitative approaches primarily involved interviews, focus groups, and exploratory design studies. This diversity reflects the exploratory and interdisciplinary nature of research at the intersection of medical AI, bias, and patient-related outcomes.

A wide range of medical AI tools was examined across the included studies (Table 3). The most frequently represented application type was conversational agents or chatbots (7/22, 31.8%), followed by LLM-based applications (4/22, 18.2%), and decision support AI (3/22, 13.6%). Diagnostic AI, robotic systems, and other ML- or NLP-based applications were each represented in 2 studies (2/22, 9.1%). One study examined a surveillance system (1/22, 4.5%), while 1 study investigated patient and public perceptions of medical AI without focusing on a specific AI tool (1/22, 4.5%).

**Table 3.** Included studies’ characteristics.

Authors	Year	Location	Study type	Publication type	AI tool	Clinical aim	AI tool purpose	AI tool function	Population
Kwak et al [45]	2024	United States	Quantitative	Retrospective cohort study	Other ML^a^ or NLP^b^-based application	Analysis of life-sustaining therapy and health equity in neurocritical care	Clinical decision support (treatment decisions)	Assessment and decision support	Adult patients with specific medical conditions (neuro-ICU^c^)
Shah et al [21]	2024	Canada	Qualitative	Methodological study or content analysis	LLM^d^	Evaluation of AI-generated patient education materials in urologic oncology	Patient education or health information	Information and education	Adult patients with cancer (urologic cancers)
Teasdale et al [46]	2024	Europe	Mixed methods	Mixed methods study	LLM	Surgical consent, patient education, trust, and informed decision-making	Patient education and informed consent support	Information and education	Mixed stakeholder sample (patients, clinicians, and staff)
Liu et al [47]	2024	United States	Mixed methods	Mixed methods study	Chatbot	Aftercare support for lung transplant recipients	Patient support and follow-up	Support, self-management, and care delivery	Adult patients with specific medical conditions (lung transplant)
Lammons et al [48]	2023	Europe	Qualitative	Qualitative focus group study	—^e^	Public and patient perspectives on AI in health care	General AI perceptions (no specific tool use)	Information and education (conceptual)	Mixed stakeholder sample (multirole participants)
Ma et al [49]	2021	United States	Quantitative	Prospective feasibility study	Chatbot	Feasibility of chatbot-based symptom monitoring in head and neck cancer	Symptom monitoring or assessment	Interaction, monitoring, and data collection	Adult patients with specific medical conditions (HNC^f^)
Carl et al [50]	2025	Europe	Quantitative	Prospective nonrandomized clinical trial	LLM	Evaluation of patient-LLM interactions in urology	Clinical decision support	Assessment and decision support	Adult clinical outpatient population
Liu et al [51]	2022	Asia	Quantitative	Quantitative cross-sectional survey study	Robot	Adoption of AI-powered service robots in hospitals	Health service automation	Support, self-management, and care delivery	General hospital patient population
Wong et al [52]	2023	Asia	Qualitative	Qualitative interview study	Surveillance system	Ethical and equity perspectives on infectious disease surveillance	Public health surveillance and ethics	Assessment and decision support	Mixed stakeholder sample (patients and clinicians)
Li and Wang [7]	2024	Asia	Quantitative	Quantitative cross-sectional study	Diagnostic AI	Determinants of AI-assisted medical service adoption	Clinical decision support (diagnostic)	Assessment and decision support	General adult hospital users (patients and relatives)
Lin et al [53]	2024	Asia	Quantitative	Quantitative cross-sectional study	Diagnostic AI	Preferences for AI-enabled community-based eye screening	Screening or diagnostic support	Assessment and decision support	General adult community population
Denecke et al [19]	2018	Europe	Mixed methods	Exploratory usability study (mixed methods)	Chatbot	Usability of digital self-anamnesis systems	Medical history collection, or anamnesis	Interaction, monitoring, and data collection	General adult nonclinical usability sample
Hong et al [54]	2022	United States	Quantitative	Observational feasibility pilot study	Chatbot	Feasibility and acceptability of AI-assisted medical interviewing	Medical history collection, or anamnesis	Interaction, monitoring, and data collection	Adult primary care patient population
Ponathil et al [55]	2020	United States	Quantitative	Primary experimental study (within-subject)	Chatbot	Usability of conversational agents for family health history collection	Medical history collection, or anamnesis	Interaction, monitoring, and data collection	General adult nonclinical volunteer sample
Nelson et al [56]	2020	United States	Qualitative	Qualitative interview study (grounded theory)	Decision support AI	Patient perceptions of AI-assisted skin cancer screening	Screening or diagnostic support	Assessment and decision support	Adult patients at risk of skin cancer
Berry et al [57]	2021	United States	Quantitative	Randomized controlled trial	Decision support AI	AI-assisted decision support for prostate cancer treatment	Clinical decision support	Assessment and decision support	Adult male patients with cancer (prostate cancer)
Zeng et al [12]	2024	Asia	Quantitative	Methodological comparative observational study	LLM	Comparison of AI- vs physician-generated patient education materials	Patient education or health information	Information and education	Mixed stakeholder sample (patients and clinicians)
Mitsea et al [58]	2023	Europe	Quantitative	Systematic review	Chatbot	AI-assisted mental health and mindfulness interventions	Behavior change or self-management support	Support, self-management, and care delivery	Mixed clinical and nonclinical adult populations
Chaix et al [20]	2019	Europe	Quantitative	Prospective observational cohort study	Chatbot	Long-term patient support via chatbot in breast cancer	Patient support and follow-up	Support, self-management, and care delivery	Adult female population with cancer (breast cancer)
Cañamero and Lewis [59]	2016	Europe	Qualitative	Qualitative exploratory design and development study	Robot	Design of a social robot for pediatric diabetes self-management	Behavior change or self-management support	Support, self-management, and care delivery	Pediatric patients with type 1 diabetes
Long et al [60]	2016	United States	Mixed Methods	Observational usability study (mixed methods)	Other ML- or NLP-based applications	AI-assisted medication reconciliation in primary care	Medical history and medication reconciliation	Interaction, monitoring, and data collection	Older adult patients with multimorbidity and polypharmacy
Kocsis et al [61]	2017	Europe	Quantitative	Methodological study or secondary data analysis	Decision support AI	ML for asthma self-management	Self-management support	Support, self-management, and care delivery	Adult patients with specific medical conditions (asthma)

a ML: machine learning.

b NLP: natural language processing.

c ICU: intensive care unit.

d LLM: large language model.

e Not available.

f HNC: head and neck cancer.

Across studies, medical AI tools were deployed for a variety of clinical aims (Table 3). The most common application was clinical decision support, including assessment, screening, and diagnostic support (8/22, 36.4%). Other frequently reported aims included long-term patient support and self-management (5/22, 22.7%), patient education and health information (4/22, 18.2%), and medical history collection or symptom monitoring (4/22, 18.2%).

Study populations predominantly consisted of adult patients (18/22, 81.8%), mostly within specific clinical contexts such as oncology, primary care, transplantation, or chronic disease management. Only 1 study focused on mental health-related medical AI applications. Several studies included mixed stakeholder samples, incorporating perspectives from patients, clinicians, or members of the general public (4/22, 18.2%). Only 1 study focused explicitly on a pediatric population.

Overall, the included studies demonstrate substantial heterogeneity in study designs, medical AI applications, clinical contexts, and target populations, highlighting the complexity of mapping bias, patient empowerment, and digital equity across medical AI research.

#### Distribution and Types of Bias Across Medical AI Applications

Across the 22 included studies, bias was predominantly described as a multilayered sociotechnical phenomenon rather than a purely technical issue. Overall, 21 (95.5%) studies reported at least one bias-related concern. Social bias was the most prevalent category in 21 of the included 22 (95.5%) studies, frequently linked to unequal representation of patient groups, health literacy and readability gaps, and language-related barriers. Algorithmic or technical bias was identified in 16 (72.7%) studies, mainly in relation to sampling and representation limitations, measurement issues, and risks of differential error patterns across subgroups. Structural bias was reported in 14 (63.6%) studies, typically reflecting broader inequities in access, digital infrastructure, and privacy or surveillance contexts that shape whether and how patients can benefit from medical AI. Design bias occurred in 12 (54.5%) studies, particularly in patient-facing interfaces where usability assumptions and default interaction designs systematically disadvantage older adults or individuals with lower digital skills. Psychological bias was less frequently coded (6/22, 27.3%) but highlighted mechanisms such as trust, automation bias effects, and algorithm aversion that can substantially influence patient reliance and engagement.

Figure 2 presents a heatmap illustrating the distribution of reported bias categories across different types of medical AI applications. Social bias was the most broadly represented bias category and occurred across the different AI application types represented in the evidence base. Algorithmic, structural, and design-related biases also occurred across several application categories, illustrating that bias was rarely confined to a single technological context. Design bias was particularly evident in patient-facing applications, including chatbots, LLM-based applications, decision support systems, and other ML- or NLP-based applications, where usability assumptions, interface design, accessibility, and interaction patterns were recurring concerns. Overall, the heatmap demonstrates that multiple bias dimensions frequently co-occurred within individual AI application types, supporting a multidimensional and sociotechnical understanding of bias in medical AI.

**Figure 2.**
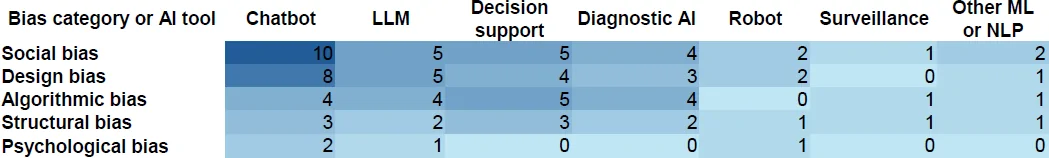
Heatmap showing the frequency of reported bias categories across different types of medical AI applications (n=22 studies). Multiple bias categories could be assigned per study; therefore, counts represent nonmutually exclusive classifications. LLM: large language model; ML: machine learning; NLP: natural language processing.

#### Distribution and Types of Patient Empowerment Domains in Medical AI Research

Empowerment-related aspects were identified across the included evidence base, although patient empowerment was rarely operationalized as a predefined outcome measure. Instead, studies frequently described empowerment-related dimensions qualitatively or inferred them from patient experiences, engagement patterns, information access, trust, autonomy, or reported benefits of AI use.

A distinction was evident between studies that explicitly sought to strengthen patients’ knowledge, participation, autonomy, or self-management and studies in which empowerment-related aspects emerged more indirectly through engagement, usability, information provision, access, trust, or technology acceptance. This distinction is important because engagement with or acceptance of an AI system does not necessarily imply increased patient control, agency, or decision-making capacity. Overall, the findings indicate that patient empowerment remains inconsistently conceptualized and operationalized across the medical AI literature.

Although empowerment-related dimensions were widely represented, many studies addressed only selected conditions of empowerment. In particular, support for knowledge acquisition and participation in care was more common than broader dimensions related to psychosocial support, everyday autonomy, or patients’ experiences of illness. These patterns suggest that empowerment is often approached through specific functional or informational dimensions rather than as a consistently defined multidimensional construct [26]. Using the thematic categorization proposed by Mora et al [23], empowerment-related findings clustered predominantly within a limited set of domains. Across the included studies, involvement in care was the most frequently represented patient empowerment domain (n=13), followed by health literacy (n=9) and trust and assurance (n=7). Health behaviors and self-management were identified in 5 studies, while illness perceptions and experiences and managing daily life were each represented in 4 studies. Perceived health was addressed in 3 studies, and social support was the least frequently represented domain (n=2; Table 4).

**Table 4.** Empowerment domains, description, and coded studies per domain. Multiple empowerment domains could be assigned to a single study.

Empowerment domain	Description	Coded studies
HL^a^	Enhancement of patients’ knowledge, understanding, access to health information, and the comprehensibility of health-related content	[7,12,19,21,46,50,54,55,60]
IC^b^	Participation in health care decisions, shared decision-making, and an active role in the care and treatment process	[7,19,21,45,46,48,51,53-57,60]
HB^c^	Support for treatment adherence, self-care practices, self-management, and health-related behavior change	[20,53,59-61]
PH^d^	Patients’ subjective perceptions of well-being, quality of life, and overall health status	[49,56,58]
IPE^e^	Understanding of illness, emotional processing, and subjective experiences related to living with a health condition	[20,57-59]
MDL^f^	Autonomy, coping with everyday life, problem-solving abilities, and functional independence	[47,58,59,61]
TA^g^	Trust in health care technologies and systems, perceived safety, and confidence in care processes and decision-making	[45-47,50-52,56]
SS^h^	Emotional, social, or community-based support experienced or facilitated through health care technologies	[20,47]

a HL: health literacy.

b IC: involvement in care.

c HB: health behaviors and self-management.

d PH: perceived health.

e IPE: illness perceptions and experiences.

f MDL: managing daily life.

g TA: trust and assurance.

h SS: social support.

Figure 3 presents a heatmap of patient empowerment domains across different types of medical AI applications. Involvement in Care was represented across nearly all application categories, whereas health literacy was concentrated primarily in LLM-based and chatbot or conversational applications. Trust and assurance were distributed across several application types, including LLM-based applications, chatbots, decision support AI, robotic systems, surveillance systems, and other ML- or NLP-based applications.

In contrast, broader psychosocial and everyday-life dimensions of empowerment, including social support, managing daily life, and illness perceptions and experiences, were represented in fewer studies and across a narrower range of AI applications. The heatmap therefore highlights both comparatively well-represented areas of the evidence base and combinations of empowerment domains and AI application types that remain sparsely represented.

**Figure 3.**
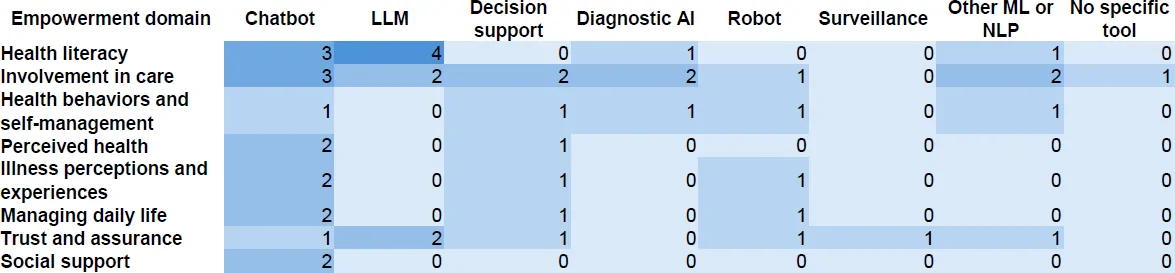
Heatmap of patient empowerment domains across medical AI application types in the included studies (n=22). Cell values indicate the number of studies in which the respective empowerment domain was identified for each AI application type. Multiple empowerment domains could be assigned to individual studies; therefore, counts are nonmutually exclusive. LLM: large language model; ML: machine learning; NLP: natural language processing.

#### Digital Divide Dimensions in Medical AI Research

Aspects of the digital divide were identified in a substantial proportion of the included studies, although they were rarely framed as a primary analytical focus. Overall, digital divide-related issues were explicitly or implicitly addressed in 19 of the 22 included studies, indicating that disparities in access, skills, or benefit from medical AI are a recurring concern across diverse applications and contexts.

Across the included literature, digital divide considerations clustered into 4 main dimensions (Table 5). The most frequently reported dimension concerned digital literacy and usability barriers, which were identified in 14 studies. These included limited technical skills, interface complexity, readability issues, and low digital confidence, particularly in patient-facing AI applications such as chatbots, LLM-based tools, self-service diagnostic systems, and mobile health (mHealth) interventions.

**Table 5.** Dimension and description of the digital divide and coded studies. Multiple digital divide dimensions could be assigned to a single study.

Digital divide dimension	Description	Studies, n (N=22)	Included studies
Digital literacy or usability	Limited technical skills, readability issues, interface complexity, low digital confidence, usability barriers	14	[7,12,19,21,46,48-51,53-57]
Age-related barriers	Older adults or children experiencing difficulties using AI-based systems	7	[19,46,49,50,52-54]
Language barriers	Language discordance, multilingual limitations, reduced comprehension of AI-generated content	4	[12,45,47,50]
Access and infrastructure	Device or internet access, geographic disparities (eg, rural vs urban), assumed constant connectivity	7	[12,20,46,48,49,57,61]

Age-related barriers were described in 7 studies, primarily affecting older adults and, in fewer cases, children. These studies reported lower familiarity with digital technologies, increased usability challenges, or greater skepticism toward AI-based systems among older users.

Language-related barriers were identified in 4 studies, highlighting reduced engagement or comprehension when AI systems were not adequately adapted to patients’ primary language or linguistic context. Language discordance was frequently associated with lower trust, reduced understanding of AI-generated information, and diminished perceived usefulness.

Finally, access- and infrastructure-related barriers were reported in 7 studies, including assumptions of stable internet connectivity, device availability, and geographic accessibility. Several studies noted disparities between urban and rural populations or emphasized that sustained use of AI-based tools presupposed resources that may not be equally available across patient groups.

Overall, these findings indicate that digital divide issues are pervasive in research on medical AI but are most often addressed implicitly. The results further suggest that digital inequities intersect closely with patient empowerment, as limitations in access, literacy, or usability can directly constrain patients’ ability to engage meaningfully with AI-supported health care, regardless of intended benefits.

To further examine how digital inequalities were distributed across medical AI contexts, digital divide dimensions were mapped against AI application types (Figure 4). Digital literacy and usability barriers were represented across the broadest range of application types, including chatbots, LLM-based applications, decision support AI, diagnostic AI, robotic systems, and studies without a specific tool. Age-related barriers were particularly represented in chatbot-, LLM-, and diagnostic AI-related studies, while language barriers occurred in a smaller subset of chatbot, LLM-based, and other ML- or NLP-based applications. Access and infrastructure barriers were distributed across chatbots, LLM-based applications, decision support AI, and studies addressing medical AI more generally. Overall, the heatmap indicates that digital inequality was not confined to a single type of medical AI application, although the specific barriers represented varied across technological contexts.

**Figure 4.**
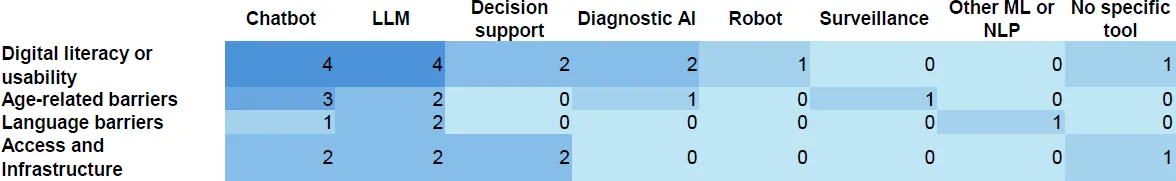
Heatmap of digital divide dimensions across medical AI application types in the included studies (n=22). Cell values indicate the number of studies addressing each combination. Multiple digital divide dimensions could be assigned to individual studies; therefore, counts are nonmutually exclusive. LLM: large language model; ML: machine learning; NLP: natural language processing.

#### Consideration of Intersectionality in Medical AI Research

Across the included studies, intersectional or multidimensional perspectives were relatively uncommon. Seven of the 22 (31.8%) included publications considered two or more social or structural dimensions of disadvantage in relation to bias, patient empowerment, or digital access. In contrast, 15 (68.2%) studies focused on individual social categories or reported demographic characteristics descriptively without considering their combined relevance.

Where intersectionality was addressed, it most commonly involved age in combination with other factors, such as digital literacy, language proficiency, or health status. For example, several studies reported that older adults with limited digital skills experienced compounded barriers when interacting with patient-facing AI systems [50,53,54]. These findings illustrate how age-related barriers may co-occur with limitations in digital literacy and other contextual factors.

A smaller subset of studies explicitly discussed race, ethnicity, or migration-related factors in combination with other social determinants. For example, Kwak et al [45] examined how race, language discordance, and neighborhood-level socioeconomic disadvantages jointly influenced AI-support decision-making in neurocritical care.

In studies addressing digital surveillance or public health AI, intersectional vulnerability was primarily discussed in ethical terms rather than empirically analyzed. Wong et al [52] highlighted that older adults, children, ethnic minorities, and sexual minorities may be disproportionately affected by AI-driven surveillance systems due to overlapping concerns related to privacy, digital literacy, and social marginalization. However, these studies did not systematically operationalize intersectionality in their analytic framework.

Overall, intersectionality was more often acknowledged as a contextual concern than operationalized as an analytical construct. Even in studies that reported detailed demographic information, analyses examining how multiple forms of disadvantage jointly relate to patient empowerment, bias exposure, or digital exclusion were largely absent.

Table 6 provides an overview of the intersectional or multidimensional dimensions identified across the included studies.

**Table 6.** Overview of studies that explicitly or implicitly addressed intersectional dimensions, including the specific combinations of social categories considered per study.

Intersectional dimension	Description	Included studies
Age × digital literacy	Older adults with limited technical skills	[50,53,54]
Race × language × SES^a^	Structural and communication-related disadvantage	[45]
Disability × access	Accessibility concerns	[46,58]
Sexual or ethnic minority × surveillance	Ethical vulnerability	[52]

a SES: socioeconomic status.

#### Debiasing Strategies

Although bias-related concerns were widely discussed across the included literature, mitigation approaches were predominantly described as recommendations or design considerations rather than as explicitly implemented and empirically evaluated debiasing strategies. Proposed approaches included improved transparency and explainability, more inclusive and representative data, participatory or user-centered design, accessibility adaptations, personalization, and stronger human oversight.

Several studies incorporated design features that could reduce bias-related barriers, such as personalization, accessible interface design, shared decision-making support, or adaptations for diverse patient groups. However, within the coding framework applied in this review, these measures were not explicitly evaluated as debiasing interventions with respect to their effects on bias, patient empowerment, or digital equity.

Overall, the evidence therefore indicates a gap between the recognition of bias in medical AI and the empirical evaluation of strategies specifically designed to mitigate its patient-related consequences.

#### Evidence Gaps Across Bias, Patient Empowerment, and Digital Equity

The descriptive mappings revealed several areas in which the identified evidence base remains comparatively sparse. With regard to patient empowerment, the literature was concentrated primarily on involvement in care, health literacy, and trust and assurance, whereas broader psychosocial and everyday-life domains, including social support, perceived health, managing daily life, and illness perceptions and experiences, were less frequently represented.

Digital divide-related factors were identified in 19 of the 22 included studies but were commonly addressed as contextual, accessibility, or usability-related concerns, rather than as primary analytical dimensions. Digital literacy and usability barriers were the most frequently represented dimensions, while language-related barriers were comparatively less frequently addressed.

Intersectional perspectives were also limited. Seven studies considered two or more social or structural dimensions of disadvantage, but such considerations remained relatively uncommon across the evidence base. Similarly, although many studies proposed or discussed approaches that could mitigate bias-related barriers, explicit empirical evaluation of debiasing strategies and their effects on patient empowerment or digital equity was largely absent.

Taken together, the evidence gaps identified in this review concern not only the number of studies addressing particular topics but also the depth with which patient empowerment, digital inequality, intersectionality, and bias mitigation are operationalized and evaluated in medical AI research.

## Discussion

### Principal Findings

This scoping review mapped how different forms of bias in medical AI are described in relation to patient empowerment and digital equity across 22 included studies. The findings indicate that bias was predominantly represented as a multidimensional sociotechnical phenomenon rather than as a purely technical problem. Social bias was the most frequently identified category, followed by algorithmic, structural, and design-related bias, with several bias dimensions frequently co-occurring within individual studies and across different medical AI application types. This pattern suggests that patient-related consequences of bias cannot be understood solely in terms of algorithmic performance but also need to be considered in relation to design choices, social conditions, and the contexts in which AI systems are implemented.

Patient empowerment was addressed across the evidence base in heterogeneous ways. Involvement in Care was the most frequently represented empowerment domain, followed by health literacy and trust and assurance, whereas broader psychosocial and everyday-life dimensions, including social support, perceived health, managing daily life, and illness perceptions and experiences, were less frequently represented. Digital divide-related factors were identified in 19 of the 22 studies, with digital literacy and usability barriers emerging most prominently. Intersectional or multidimensional dimensions of disadvantage were considered in 7 studies but remained comparatively uncommon. Similarly, while many studies discussed potential approaches to reducing bias-related barriers, explicit empirical evaluation of debiasing strategies in relation to patient empowerment or digital equity was largely absent.

Taken together, these findings show that current research recognizes many of the social and patient-related implications of bias in medical AI, but these dimensions are not yet addressed with equal conceptual or methodological depth. The evidence base is particularly concentrated on participation, information-related dimensions of empowerment, trust, and usability, while broader dimensions of empowerment, intersectional disadvantage, and empirically evaluated bias mitigation remain less developed. By integrating bias, patient empowerment, and digital equity within a single analytical perspective, this review therefore highlights both the multidimensional nature of bias in medical AI and important gaps in how its patient-related consequences are currently conceptualized and evaluated.

### Interpretation of Findings and Comparison With Prior Work

#### Bias in Medical AI as a Sociotechnical Phenomenon

Consistent with recent reviews on bias in medical LLMs and health care AI more broadly, the reviewed studies suggest that bias in medical AI cannot be understood solely as a technical performance issue [34,35]. Existing reviews have primarily emphasized demographic performance disparities, data imbalance, algorithmic fairness, and mitigation strategies [34,35,62]. However, our synthesis extends this perspective by showing how bias becomes relevant for patient empowerment through design choices, usability assumptions, language accessibility, trust, and structural conditions of deployment. Across the included studies, bias emerged across multiple sociotechnical layers throughout the AI lifecycle, including data practices, algorithmic design, user interfaces, institutional contexts, and human-AI interaction [27,28,41].

While algorithmic biases, including concerns related to sampling and unequal performance across demographic groups, were frequently discussed in the included studies [45,57,61], our results highlight the relevance of design and structural bias for patient empowerment. This finding is in line with other research in this area emphasizing how interface design, usability assumptions, and default interaction patterns may privilege certain users while disadvantaging others [41,43].

In this scoping review, design bias was identified across several patient-facing medical AI application types [19,53,55]. These systems often assumed a baseline level of digital literacy, language proficiency, and physical or cognitive ability, thereby potentially reproducing existing inequities rather than mitigating them. As argued by Leslie [27] and Cross et al [3], such biases are not incidental but reflect deeper structural conditions in which AI systems are developed and deployed.

#### Patient Empowerment

An important contribution of this scoping review is the attempt to clearly distinguish patient empowerment as an explicit research goal from empowerment-related aspects that emerge as a by-product of AI implementation. Because the inclusion criteria encompassed both explicit and implicit relevance to patient empowerment, empowerment was not necessarily conceptualized or operationalized as a distinct outcome across the included studies. Instead, empowerment-related aspects were frequently reflected in indicators such as engagement, satisfaction, trust, or technology acceptance.

This pattern mirrors concerns raised in previous work on patient empowerment, which has shown that the concept is frequently used inconsistently and measured with heterogeneous instruments [17,22,23,63]. The literature reviewed in this study suggests that this conceptual ambiguity persists in medical AI research, where empowerment is often inferred from engagement, satisfaction, trust, usability, or information access rather than examined as a multidimensional construct involving knowledge, control, participation, and evaluative capacity, as originally described by Funnell et al [26]. By explicitly distinguishing between empowerment as a conceptual condition and empowerment-related domains as descriptive categories, this review advances prior work that has primarily documented definitional heterogeneity. Our findings indicate that medical AI research frequently approaches empowerment through engagement, information provision, or technology acceptance [17,63]. Without sufficient support for patient control and evaluative capacity [26], such interventions may result in a form of “thin” or superficial empowerment that may coexist with persistent inequities.

By applying the thematic categorization proposed by Mora et al [23], our analysis makes visible which dimensions of patient empowerment are prioritized in current medical AI research and which are less frequently represented. Involvement in Care was the most frequently represented domain, followed by health literacy and trust and assurance. These domains were particularly evident in AI-based patient education [12,21], decision support systems [57], and LLM-based information tools [50]. These findings align with the broader digital health literature, which has long emphasized information access and shared decision-making as core components of patient empowerment [63].

In contrast, broader psychosocial and everyday-life domains, including social support, perceived health, managing daily life, and illness perceptions and experiences, were less frequently represented. When present, these domains were primarily identified in long-term condition management, oncology support chatbots, and mental health-related applications [20,58,59]. This imbalance may suggest that patient empowerment in medical AI is predominantly framed in informational and participatory terms, while broader psychosocial, relational, and everyday-life dimensions remain comparatively underexplored.

#### Digital Divide as Condition for Patient Empowerment

Across the reviewed literature, the digital divide consistently emerged as a key factor shaping whether patients could meaningfully benefit from AI-supported health care. These findings align with prior reviews on digital health disparities, which emphasize that digital inequality cannot be reduced to access alone and that intersectional dimensions of disadvantage remain insufficiently theorized in digital health research [31-33]. Our review extends this argument to medical AI by showing that digital divide mechanisms are often embedded in AI design and deployment contexts, for example, through assumptions about literacy, language proficiency, usability, and continuous connectivity. Across the included studies, disparities were most commonly discussed in relation to digital literacy, age, language proficiency, and familiarity with digital tools. Several studies reported that older adults and individuals with limited technical skills faced greater barriers when interacting with AI-based systems, particularly chatbots, self-service diagnostics, and mHealth applications [49,53,60]. Language barriers were also repeatedly identified, both in patient education tools [12] and in clinical contexts where language discordance undermined trust and comprehension [45].

Importantly, our results indicate that the digital divide is not merely an external constraint but can also be reinforced through design choices, linking it closely to design bias [33]. This observation strengthens the argument that patient empowerment-oriented medical AI should explicitly integrate digital inclusion principles, rather than assuming that technological availability translates into meaningful use.

Our findings further suggest that digital inclusion deficits may limit the extent to which patient engagement translates into patient empowerment. While many AI-based interventions increase interaction, information exposure, or technology use, these forms of engagement do not necessarily translate into empowerment in the sense proposed by Funnell et al [26], that is, if patients lack the resources, control, or evaluative capacity to meaningfully act on AI-supported recommendations. In this way, digital divide mechanisms may create the appearance of empowerment at the surface level while constraining some of its foundational conditions.

Our results support a shift away from viewing the digital divide as a simple access issue. In the included evidence base, barriers related to digital literacy and usability were more frequently represented than access and infrastructure barriers, while language-related barriers were less frequently addressed. This suggests that empowerment-related benefits may depend not only on access to AI-supported health care but also on patients’ ability to meaningfully engage with AI-mediated information and interactions. Importantly, the findings also resonate with the notion of adverse digital incorporation [32]: patients may be included in AI-enabled care on unequal terms, for example, through interfaces that demand high literacy, consent processes that are difficult to understand, or deployments that intensify data capture and surveillance, thereby potentially constraining autonomy and trust. In this sense, digital inclusion is not inherently empowering; under unequal conditions, it may create new dependencies or shift burdens to patients who are less equipped to negotiate them. High uptake or engagement, therefore, does not necessarily imply increased control, understanding, or decision capacity [17,63]. Empowerment-sensitive medical AI should consequently integrate digital inclusion principles at the level of design and governance, rather than treating them solely as downstream implementation concerns.

#### Intersectionality: Persistent Blind Spots

Although several studies considered intersectional or multidimensional dimensions of disadvantage, such perspectives remained relatively uncommon across the included evidence base. Most studies focused on single social or structural dimensions, such as age, race, education, or digital literacy, without examining their combined relevance for patients’ experiences of AI systems.

This finding is consistent with previous work highlighting the limited integration of intersectionality in digital health disparity research [36] and the underrepresentation of diverse or marginalized populations in AI-supported health and social care [37]. More broadly, intersectionality theory emphasizes that social positions and structures of disadvantage cannot always be adequately understood in isolation [64,65]. Our findings add that, even when included studies considered multiple dimensions of disadvantage, these dimensions were rarely integrated analytically in relation to patient empowerment or AI-mediated digital inclusion. Studies such as Wong et al [52] and Kwak et al [45], for example, illustrate how multiple dimensions of social and structural disadvantage may be relevant within medical AI contexts without necessarily being examined through an explicit intersectional analytical framework.

The limited integration of intersectional approaches therefore represents a persistent blind spot in current medical AI research and constrains the ability to understand how different dimensions of disadvantage may jointly shape bias exposure, patient empowerment, and digital exclusion. More systematic consideration of intersectionality represents an important direction for future research.

#### Debiasing Strategies: From Awareness to Action

While bias was widely recognized across the literature, mitigation approaches were predominantly discussed as recommendations, design considerations, or future directions rather than as explicitly implemented and empirically evaluated debiasing strategies. This finding resonates with broader work emphasizing the need to address bias systematically across the AI lifecycle, from model conception and development through deployment and postdeployment monitoring [35], and with established fairness and AI ethics frameworks highlighting the importance of translating principles into practice [27,28].

Our findings further indicate that mitigation approaches were rarely evaluated specifically in relation to patient empowerment or digital inclusion. This is particularly relevant from a patient-centered perspective, as human-centered AI approaches emphasize the importance of autonomy, trust, communication, and meaningful patient involvement when evaluating AI-supported health care [25]. Fairness-oriented evaluation may therefore benefit from considering not only technical performance metrics but also patient-centered outcomes, accessibility, and meaningful participation.

Several studies incorporated design features that could reduce bias-related barriers, including personalization, participatory or user-centered design elements, accessibility adaptations, shared decision-making support, and improved transparency. However, these measures were generally not evaluated explicitly as debiasing interventions with respect to their effects on bias, patient empowerment, or digital equity. This gap highlights the need for more empirically grounded evaluation of mitigation strategies that consider both technical fairness and patient-related consequences.

#### Integrative Mapping: Bias, Digital Divide, and Patient Empowerment

Building on the cross-domain patterns identified in the evidence base, Figure 5 provides an integrative conceptual representation of the relationships between bias, digital inequality, and patient empowerment. Based on the synthesis of the included studies, the findings suggest that bias, digital inequality, and empowerment-related outcomes are closely interconnected across the lifecycle of patient-facing medical AI systems.

Bias, particularly in terms of design and structural conditions, may shape who can access, understand, and trust medical AI systems. Such biases may contribute to digital inequalities not only in terms of access but also through usability, comprehension, and the ability to realize potential benefits from AI-supported care [31-33,41]. Digital inequalities may, in turn, constrain the extent to which medical AI supports patient empowerment rather than merely increasing engagement [17,26,63]. Patient empowerment may subsequently shape trust, adoption, and the longer-term integration of AI systems into care practices, suggesting potential feedback relationships between patient experiences, system use, and future design and deployment.

**Figure 5.**
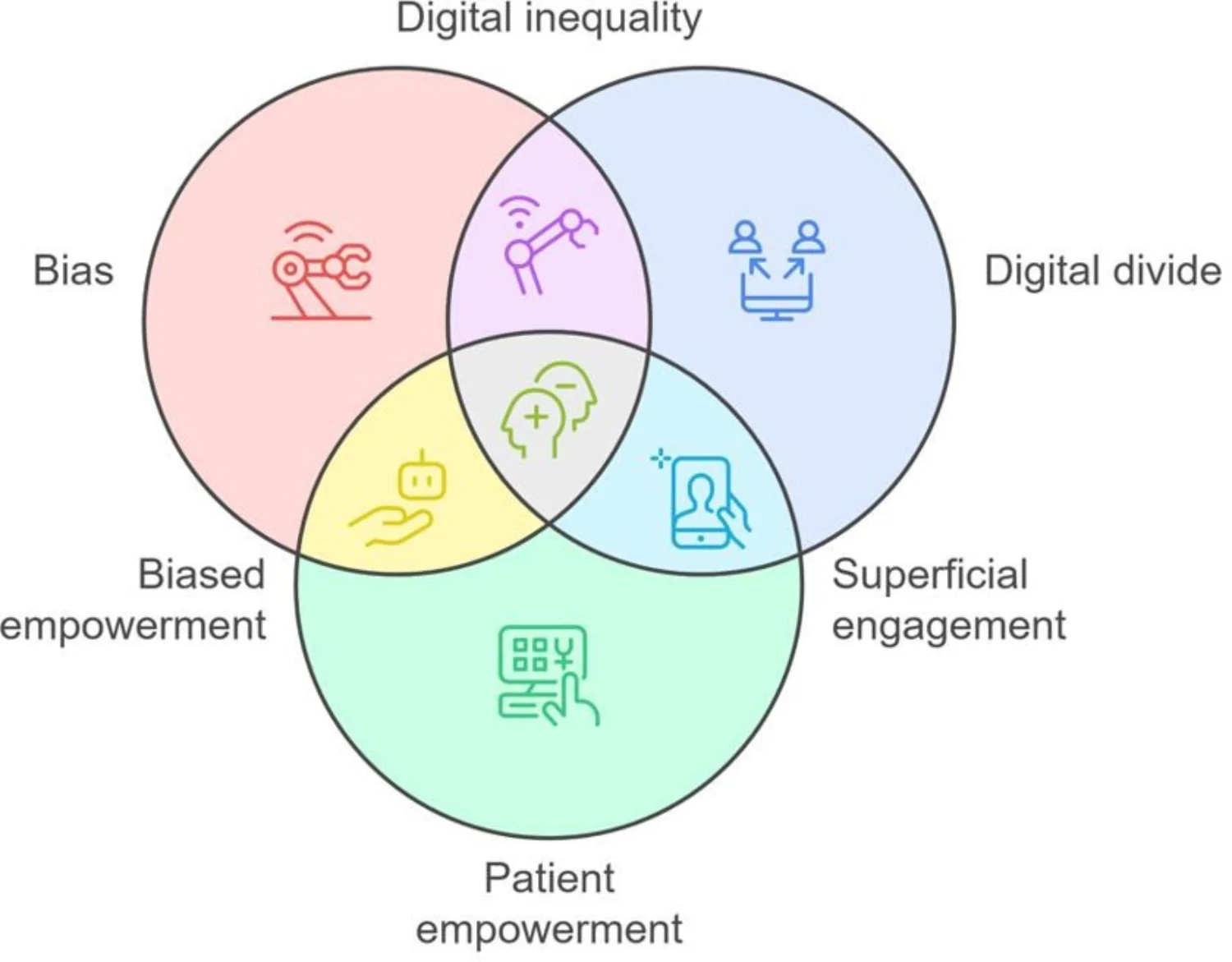
The interplay of bias, digital divide, and patient empowerment.

### Implications for Research and Design: Operationalizing Intersectionality and Empowerment in Medical AI

The findings of this scoping review highlight the need to move beyond primarily conceptual discussions of equity and patient empowerment toward more operational and empirically applicable approaches in medical AI research and design. In particular, intersectionality and patient empowerment should not be treated merely as abstract ethical aspirations, but as methodological and design considerations that can inform how AI systems are developed, evaluated, and implemented [25,36].

First, future studies should more systematically consider multiple social and structural dimensions of disadvantage, such as age, gender, ethnicity, socioeconomic status, and digital literacy, where theoretically and methodologically appropriate, rather than examining these dimensions exclusively in isolation [36,64,65]. This may help clarify how overlapping forms of disadvantage shape access to, interaction with, and outcomes of AI-supported care.

Second, studies should more explicitly distinguish patient engagement from patient empowerment. Empowerment-oriented medical AI should not only increase interaction or information exposure, but also support patients’ understanding, evaluative capacity, and ability to exercise meaningful control in AI-supported decisions [17,26,63].

Third, the findings underscore the importance of integrating equity and inclusion considerations directly into the design process of medical AI systems. Participatory and co-design approaches involving diverse patient groups, including those experiencing social or digital disadvantage, may help identify design-related barriers and improve meaningful access and usability [25].

Finally, improved reporting of sociodemographic characteristics, contextual factors, and design assumptions is essential for enabling intersectional interpretation and cross-study comparability in medical AI research [35,37].

The findings of this review are also relevant in light of evolving AI governance frameworks in health care, including the European Union AI Act [66]. This regulatory framework emphasizes transparency, data governance, human oversight, and lifecycle management of AI-enabled systems. However, the present findings indicate that patient empowerment, digital inclusion, and intersectional dimensions of disadvantage remain comparatively underdeveloped within the identified medical AI literature. More explicit consideration of these dimensions in evaluation, design, and governance may therefore contribute to more equitable and patient-centered implementation of AI-enabled health care.

### Limitations

This scoping review has several limitations that should be considered when interpreting the findings. First, the literature included was highly heterogeneous with respect to study designs, AI applications, clinical contexts, and reported outcomes. This heterogeneity limited the comparability of findings and precluded quantitative synthesis or robust cross-study comparisons, particularly regarding empowerment-related outcomes.

Second, no formal methodological quality appraisal or risk-of-bias assessment of the included studies was conducted. Such an assessment was not part of the mapping-oriented objective of this scoping review, which focused on conceptualizations, thematic patterns, and evidence gaps, rather than on estimating intervention effectiveness or the certainty of evidence. Consequently, the findings should be interpreted as descriptive patterns within the identified evidence base rather than as evidence of causal effects or effectiveness.

Third, although the search strategy was iteratively refined in line with the mapping-oriented purpose of the review, it may not have captured all potentially relevant literature. The strategy prioritized studies at the intersection of medical AI, bias, and patient-related implications, and relevant studies addressing equity, digital inclusion, or empowerment only indirectly may therefore have been missed. In addition, the search focused primarily on biomedical, psychological, and health services databases and did not include major multidisciplinary and engineering-oriented sources such as IEEE Xplore, Scopus, or Web of Science. Consequently, the resulting evidence map should be interpreted as a structured mapping of the identified patient-centered literature rather than as evidence that all potentially relevant research on bias in medical AI has been captured.

Finally, there is a risk of overinterpreting patient empowerment in studies where empowerment-related aspects were inferred indirectly from measures such as technology acceptance, satisfaction, or engagement. Increased use of or positive attitudes toward medical AI do not necessarily translate into meaningful empowerment, particularly when patients’ control, resources, understanding, or evaluative capacities are not explicitly assessed. The empowerment-related findings should therefore be interpreted as thematic mapping of potentially relevant dimensions rather than as evidence that the included AI applications produced patient empowerment.

### Conclusions

This scoping review highlights that bias in medical AI is not merely a technical issue but is closely intertwined with patient empowerment and digital inclusion. Across the identified evidence base, bias was represented across multiple sociotechnical dimensions, while empowerment-related research was concentrated primarily on involvement in care, health literacy, and trust and assurance. Broader psychosocial and everyday-life dimensions of empowerment were less frequently represented.

The findings further indicate that digital inequalities extend beyond access to include barriers related to digital literacy, usability, language, and the ability to benefit meaningfully from AI-supported health care. Intersectional or multidimensional dimensions of disadvantage were considered in a minority of studies, and mitigation approaches were predominantly discussed as recommendations or design considerations rather than explicitly evaluated as debiasing interventions. Future research should therefore more systematically consider multiple forms of disadvantage, distinguish patient engagement from patient empowerment, and evaluate how design and mitigation approaches affect patient-related outcomes and digital equity.

By linking bias, patient empowerment, and digital inclusion within a single analytical perspective, this review extends fairness-oriented medical AI research beyond technical performance considerations alone. The findings suggest that equitable and trustworthy medical AI will require attention not only to algorithmic bias, but also to inclusive design, accessibility, governance, and meaningful patient participation across the AI lifecycle. Without such considerations, AI-supported health care may reproduce existing inequalities while offering only limited opportunities for meaningful patient empowerment.

## Supplementary material

10.2196/94439 Multimedia Appendix 1 Full search strategies and search process documentation.

10.2196/94439 Multimedia Appendix 2 Reference list of included papers.

10.2196/94439 Multimedia Appendix 3 Data extraction of 22 studies.

10.2196/94439 Checklist 1 PRISMA-ScR Checklist.

## Abbreviations

CDSS: clinical decision support system
JBI: Joanna Briggs Institute
LLM: large language model
mHealth: mobile health
ML: machine learning
NLP: natural language processing
OSF: Open Science Framework
PCC: population-concept-context
PRISMA: Preferred Reporting Items for Systematic Reviews and Meta-Analyses
PRISMA-S: Preferred Reporting Items for Systematic Reviews and Meta-Analyses literature search extension
PRISMA-ScR: Preferred Reporting Items for Systematic Reviews and Meta-Analyses extension for Scoping Reviews
PROSPERO: International Prospective Register of Systematic Reviews
WHO: World Health Organization
