# Supplementary material for: Bias in Medical AI in Relation to Patient Empowerment and Digital Equity: Scoping Review
Source: Online J Public Health Inform. 2026 Nov 9;18:e94439. doi: 10.2196/94439 (PMC13747314; doi:10.2196/94439)
Supplement: Supplementary file 2 [file ojphi-v18-e94439-s001.docx]

Literature Search Strategy

The literature search was conducted in August 2025 using PubMed/MEDLINE and EBSCOHost databases. The search strategy was developed iteratively based on pilot searches and refined to balance conceptual breadth with the patient-centered focus of the review.

The search process initially included an explicit digital divide search block. However, pilot searches demonstrated that this approach substantially restricted retrieval and excluded studies addressing digital divide-related dimensions only implicitly (e.g., through usability barriers, language accessibility, or differential benefit from AI-supported healthcare). Consequently, the explicit digital divide block was removed during search refinement prior to full screening and data extraction. All records identified during the initial searches were retained for screening.

The final search strategy combined concepts related to:

- Medical artificial intelligence and digital health technologies,
- Bias, fairness, inequity, and discrimination,
- Patient empowerment, participation, and involvement.

Search strategies were adapted to the syntax and indexing systems of each database. Both controlled vocabulary terms (e.g., MeSH terms) and free-text keywords were used where applicable.

PubMed/MEDLINE Search Strategy

(

  "Artificial Intelligence"[Mesh] OR "Machine Learning"[Mesh] OR "Natural Language Processing"[Mesh] OR "Deep Learning"[Mesh] OR "Clinical Decision Support Systems"[Mesh] OR "Telemedicine"[Mesh] OR

  "artificial intelligence"[tiab] OR "machine learning"[tiab] OR "natural language processing"[tiab] OR "deep learning"[tiab] OR "clinical decision support"[tiab] OR "healthcare AI"[tiab] OR "large language model*"[tiab] OR LLM*[tiab] OR ehealth[tiab] OR "e-health"[tiab] OR telehealth[tiab] OR "tele-health"[tiab]

)

AND

(

  "Prejudice"[Mesh] OR "Healthcare Disparities"[Mesh] OR

  bias*[tiab] OR inequit*[tiab] OR inequal*[tiab] OR discriminat*[tiab] OR fair*[tiab] OR intersectional*[tiab]

)

AND

(

  "Patient Participation"[Mesh] OR "Decision Making, Shared"[Mesh] OR

  "patient empowerment"[tiab] OR "shared decision*"[tiab] OR "patient participation"[tiab] OR "patient inclusion"[tiab] OR "patient engagement"[tiab] OR "patient involvement"[tiab]

)

PubMed Search Results

Initial pilot search including digital divide block: 26 records

Refined search without explicit digital divide block: 239 records

EbscoHost Search Strategy

Databases included within EBSCOHost:

- MEDLINE
- APA PsycInfo
- APA PsycArticles
- ERIC
- CINAHL Ultimate
- Academic Search Elite
- Additional social science and information science databases available through the institutional EBSCOHost platform

(TI("artificial intelligence" OR "machine learning" OR "natural language processing" OR "deep learning" OR "clinical decision support" OR "healthcare AI" OR "large language model*" OR LLM* OR ehealth OR "e-health" OR telehealth OR "tele-health")

OR AB("artificial intelligence" OR "machine learning" OR "natural language processing" OR "deep learning" OR "clinical decision support" OR "healthcare AI" OR "large language model*" OR LLM* OR ehealth OR "e-health" OR telehealth OR "tele-health")

OR SU("artificial intelligence" OR "machine learning" OR "natural language processing" OR "deep learning" OR "clinical decision support" OR "healthcare AI" OR "large language model*" OR LLM* OR ehealth OR "e-health" OR telehealth OR "tele-health"))

AND

(TI(bias* OR inequit* OR inequal* OR discriminat* OR fair* OR intersectional*)

OR AB(bias* OR inequit* OR inequal* OR discriminat* OR fair* OR intersectional*)

OR SU(bias* OR inequit* OR inequal* OR discriminat* OR fair* OR intersectional*))

AND

(TI("patient empowerment" OR "shared decision*" OR "patient participation" OR "patient inclusion" OR "patient engagement" OR "patient involvement")

OR AB("patient empowerment" OR "shared decision*" OR "patient participation" OR "patient inclusion" OR "patient engagement" OR "patient involvement")

OR SU("patient empowerment" OR "shared decision*" OR "patient participation" OR "patient inclusion" OR "patient engagement" OR "patient involvement"))

EBSCOHost Search Results

Initial pilot search including digital divide block: 20 records

Refined search without explicit digital divide block: 249 records

Search Refinement and Screening Process

Pilot searches demonstrated that requiring explicit digital divide terminology substantially reduced sensitivity and excluded studies in which digital inequities were discussed implicitly rather than as primary analytical categories.

During initial abstract screening 46 records were screened, including 5 duplicate records, resulting in 41 unique abstracts assessed.

Inter-reviewer agreement during pilot screening was approximately 88%.

Following refinement of the search strategy PubMed yielded 239 records, EBSCOHost yielded 249 records.

After duplicate removal, 255 unique records remained for title and abstract screening.

All records were imported into Rayyan for duplicate removal, blinded screening, and screening management.

Additional Search Procedures

Google Scholar was used for supplementary searching to identify potentially relevant additional publications. No additional eligible studies meeting the inclusion criteria were identified through supplementary searching.

Reference list screening of included studies and relevant review articles was additionally performed to identify potentially relevant literature not captured through database searching.

Search Limits

The following eligibility limits were applied during screening:

- Publication year: 2015 onwards
- Language: English or German
- Publication type: Peer-reviewed journal articles

No geographic restrictions were applied.
