# Supplementary material for: Bias in Medical AI in Relation to Patient Empowerment and Digital Equity: Scoping Review
Source: Online J Public Health Inform. 2026 Nov 9;18:e94439. doi: 10.2196/94439 (PMC13747314; doi:10.2196/94439)
Supplement: Supplementary file 3 [file ojphi-v18-e94439-s002.docx]

56. Berry DL, Hong F, Blonquist TM, Halpenny B, Xiong N, Filson CP, et al. Decision regret, adverse outcomes, and treatment choice in men with localized prostate cancer: Results from a multi-site randomized trial. Urologic Oncology: Seminars and Original Investigations 2021;39:493.e9-493.e15. https://doi.org/10.1016/j.urolonc.2020.11.038.

58. Cañamero L, Lewis M. Making New “New AI” Friends: Designing a Social Robot for Diabetic Children from an Embodied AI Perspective. Int J Soc Robot 2016;8:523–37. https://doi.org/10.1007/s12369-016-0364-9.

49. Carl N, Haggenmüller S, Wies C, Nguyen L, Winterstein JT, Hetz MJ, et al. Evaluating interactions of patients with large language models for medical information. BJU Int 2025;135:1010–7. https://doi.org/10.1111/bju.16676.

20. Chaix B, Bibault JE, Pienkowski A, Delamon G, Guillemassé A, Nectoux P, et al. When chatbots meet patients: One-year prospective study of conversations between patients with breast cancer and a chatbot. JMIR Cancer 2019;5:1–7. https://doi.org/10.2196/12856.

19. Denecke K, Hochreutener SL, Pöpel A, May R. Self-Anamnesis with a Conversational User Interface: Concept and Usability Study. Methods Inf Med 2018;57:243–52. https://doi.org/10.1055/s-0038-1675822.

53. Hong G, Smith M, Lin S. The AI Will See You Now: Feasibility and Acceptability of a Conversational AI Medical Interviewing System. JMIR Form Res 2022;6:1–6. https://doi.org/10.2196/37028.

60. Kocsis O, Arvanitis G, Lalos A, Moustakas K, Sont JK, Honkoop PJ, et al. Assessing machine learning algorithms for self-management of asthma. 2017 E-Health and Bioengineering Conference, EHB 2017 2017:571–4. https://doi.org/10.1109/EHB.2017.7995488.

44. Kwak GH, Kamdar HA, Douglas MJ, Hu H, Ack SE, Lissak IA, et al. Social Determinants of Health and Limitation of Life-Sustaining Therapy in Neurocritical Care: A CHoRUS Pilot Project. Neurocrit Care 2024;41:866–79. https://doi.org/10.1007/s12028-024-02007-0.

47. Lammons W, Silkens M, Hunter J, Shah S, Stavropoulou C. Centering Public Perceptions on Translating AI Into Clinical Practice: Patient and Public Involvement and Engagement Consultation Focus Group Study. J Med Internet Res 2023;25. https://doi.org/10.2196/49303.

7. Li W, Wang J. Determinants of artificial intelligence-assisted diagnostic system adoption intention: A behavioral reasoning theory perspective. Technol Soc 2024;78:102643. https://doi.org/10.1016/j.techsoc.2024.102643.

52. Lin S, Ma Y, Jiang Y, Li W, Peng Y, Yu T, et al. Service Quality and Residents’ Preferences for Facilitated Self-Service Fundus Disease Screening: Cross-Sectional Study. J Med Internet Res 2024;26:1–13. https://doi.org/10.2196/45545.

46. Liu AW, Brown W, Madu NE, Maiorano AR, Bigazzi O, Medina E, et al. Patient Engagement With and Perspectives on a Mobile Health Home Spirometry Intervention: Mixed Methods Study. JMIR Mhealth Uhealth 2024;12. https://doi.org/10.2196/51236.

50. Liu X, He X, Wang M, Shen H. What influences patients’ continuance intention to use AI-powered service robots at hospitals? The role of individual characteristics. Technol Soc 2022;70:101996. https://doi.org/10.1016/j.techsoc.2022.101996.

59. Long J, Yuan MJ, Poonawala R. An Observational Study to Evaluate the Usability and Intent to Adopt an Artificial Intelligence–Powered Medication Reconciliation Tool. Interact J Med Res 2016;5:e14. https://doi.org/10.2196/ijmr.5462.

48. Ma D, Orner D, Ghaly MM, Parashar B, Ames JW, Chen WC, et al. Automated health chats for symptom management of head and neck cancer patients undergoing radiation therapy. Oral Oncol 2021;122. https://doi.org/10.1016/j.oraloncology.2021.105551.

57. Mitsea E, Drigas A, Skianis C. Digitally Assisted Mindfulness in Training Self-Regulation Skills for Sustainable Mental Health: A Systematic Review. Behavioral Sciences 2023;13. https://doi.org/10.3390/bs13121008.

55. Nelson CA, Pérez-Chada LM, Creadore A, Li SJ, Lo K, Manjaly P, et al. Patient Perspectives on the Use of Artificial Intelligence for Skin Cancer Screening. JAMA Dermatol 2020;156:501. https://doi.org/10.1001/jamadermatol.2019.5014.

54. Ponathil A, Ozkan F, Welch B, Bertrand J, Chalil Madathil K. Family health history collected by virtual conversational agents: An empirical study to investigate the efficacy of this approach. J Genet Couns 2020;29:1081–92. https://doi.org/10.1002/jgc4.1239.

21. Shah YB, Ghosh A, Hochberg A, Mark JR, Lallas CD, Shah MS. Artificial intelligence improves urologic oncology patient education and counseling. 2024.

45. Teasdale A, Mills L, Costello R. Artificial Intelligence-Powered Surgical Consent: Patient Insights. Cureus 2024;16:1–11. https://doi.org/10.7759/cureus.68134.

51. Wong WCW, Zhao IY, Ma YX, Dong WN, Liu J, Pang Q, et al. Primary Care Physicians’ and Patients’ Perspectives on Equity and Health Security of Infectious Disease Digital Surveillance. Ann Fam Med 2023;21:33–9. https://doi.org/10.1370/afm.2895.

12. Zeng S, Kong Q, Wu X, Ma T, Wang L, Xu L, et al. Artificial Intelligence-Generated Patient Education Materials for Helicobacter pylori Infection: A Comparative Analysis. Helicobacter 2024;29:1–10. https://doi.org/10.1111/hel.13115.
